# Bioinspired MXene-Based Soft Actuators Exhibiting Angle-Independent Structural Color

**DOI:** 10.1007/s40820-022-00977-4

**Published:** 2022-11-28

**Authors:** Pan Xue, Yuanhao Chen, Yiyi Xu, Cristian Valenzuela, Xuan Zhang, Hari Krishna Bisoyi, Xiao Yang, Ling Wang, Xinhua Xu, Quan Li

**Affiliations:** 1 School of Materials Science and Engineering, Tianjin University, Tianjin, 300350 People’s Republic of China; 2 Institute of Advanced Materials, School of Chemistry and Chemical Engineering, Tech Key Laboratory for Biomedical Research, Southeast University, and Jiangsu Province Hi, Nanjing, 211189 People’s Republic of China; 3 Advanced Materials and Liquid Crystal Institute and Chemical Physics Interdisciplinary Program, Kent State University, Kent, OH 44242 USA

**Keywords:** Bioinspired soft actuator, Angle-independent structural color, MXene liquid crystals, Soft robotics

## Abstract

In nature, many living organisms exhibiting unique structural coloration and soft-bodied actuation have inspired scientists to develop advanced structural colored soft actuators toward biomimetic soft robots. However, it is challenging to simultaneously biomimic the angle-independent structural color and shape-morphing capabilities found in the plum-throated cotinga flying bird. Herein, we report biomimetic MXene-based soft actuators with angle-independent structural color that are fabricated through controlled self-assembly of colloidal SiO_2_ nanoparticles onto highly aligned MXene films followed by vacuum-assisted infiltration of polyvinylidene fluoride into the interstices. The resulting soft actuators are found to exhibit brilliant, angle-independent structural color, as well as ultrafast actuation and recovery speeds (a maximum curvature of 0.52 mm^−1^ can be achieved within 1.16 s, and a recovery time of ~ 0.24 s) in response to acetone vapor. As proof-of-concept illustrations, structural colored soft actuators are applied to demonstrate a blue gripper-like bird’s claw that can capture the target, artificial green tendrils that can twine around tree branches, and an artificial multicolored butterfly that can flutter its wings upon cyclic exposure to acetone vapor. The strategy is expected to offer new insights into the development of biomimetic multifunctional soft actuators for somatosensory soft robotics and next-generation intelligent machines.

**Supplementary Information:**

The online version contains supplementary material available at 10.1007/s40820-022-00977-4.

## Highlights

- Design and fabrication of MXene-based soft actuators with angle-independent structural color.
- The nanostructured MXene can not only facilitate the formation of short-range ordered 3D amorphous photonic crystals, but also help significantly improve structural color saturation.
- The soft actuators exhibit brilliant angle-independent structural color, ultrafast actuation and recovery speeds in response to vapor.

**Supplementary Information:**

The online version contains supplementary material available at 10.1007/s40820-022-00977-4.

## Graphical abstract

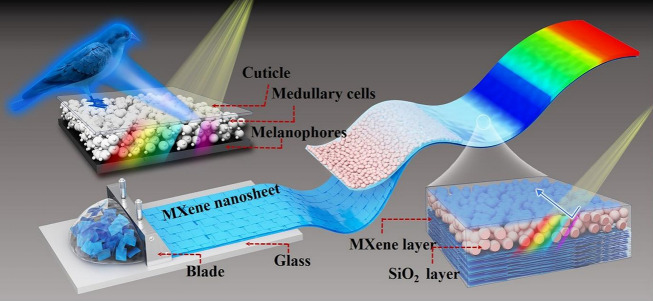

## Introduction

Many living organisms possess the unique capability of simultaneously actuating their soft bodies and changing their skin colors [1–6]. For example, chameleons are capable of rapid body locomotion to escape threats from predators and of adaptively changing skin color to camouflage themselves by altering the lattice of guanine nanocrystals within the superficial skin layer of dermal iridophores [7]. Many birds, such as the plum-throated cotinga in nature, can not only flap their wings with the muscles to fly and glide freely in the air but also display brilliant blue feathers with angle-independent structural colors, which are believed to result from the disordered or short-range ordered three-dimensional (3D) amorphous photonic nanostructures enhanced by underlying black melanin particles [8, 9]. Taking inspiration from nature, researchers have devoted extensive efforts to developing artificial structurally colored soft actuators that exhibit biomimetic color functionality and programmable shape transformations in response to external stimuli [10–15]. For example, inspired by the molecular channels that widely exist in living cells and tissue, humidity-driven structurally colored soft actuators with multicolor switching capability were conceptually demonstrated by integrating chromogenic photonic crystals into intrinsically deformable soft materials [11]. Taking lessons from the adaptive color regulation mechanism of chameleons, vapor-driven structurally colored soft actuators exhibiting both biomimetic color changes and programmable shape transformations were developed by introducing patterned polymer stripes into synthetic inverse opal films [12]. Furthermore, bioinspired, living, structurally colored soft actuators were reported by assembling engineered cardiomyocyte tissues onto synthetic inverse opal hydrogels, where autonomous shape and color regulation capability was enabled by the cell contraction and elongation in the beating processes of the cardiomyocytes [15]. Compared to chemically colored soft actuators loaded with dyes or pigments [16–19], physically colored (structurally colored) soft actuators integrated with photonic crystals display brilliant colors that are dynamically tunable by adjusting the lattice constant or refractive index and never fade as long as the periodic structures persist [20–25]. However, the structural color of soft actuators reported thus far is dependent on the viewing and light illumination angles, which is one of the critical limitations compared with chemically colored soft actuators. It should be noted that angle-independent structural colors are truly useful for industry because they look a lot like dyes and pigments, but they do not need to absorb any light. We could imagine making structurally colored soft actuators with constant color in any observation angle under the sun because its reflecting the light.

**Fig. 1:**
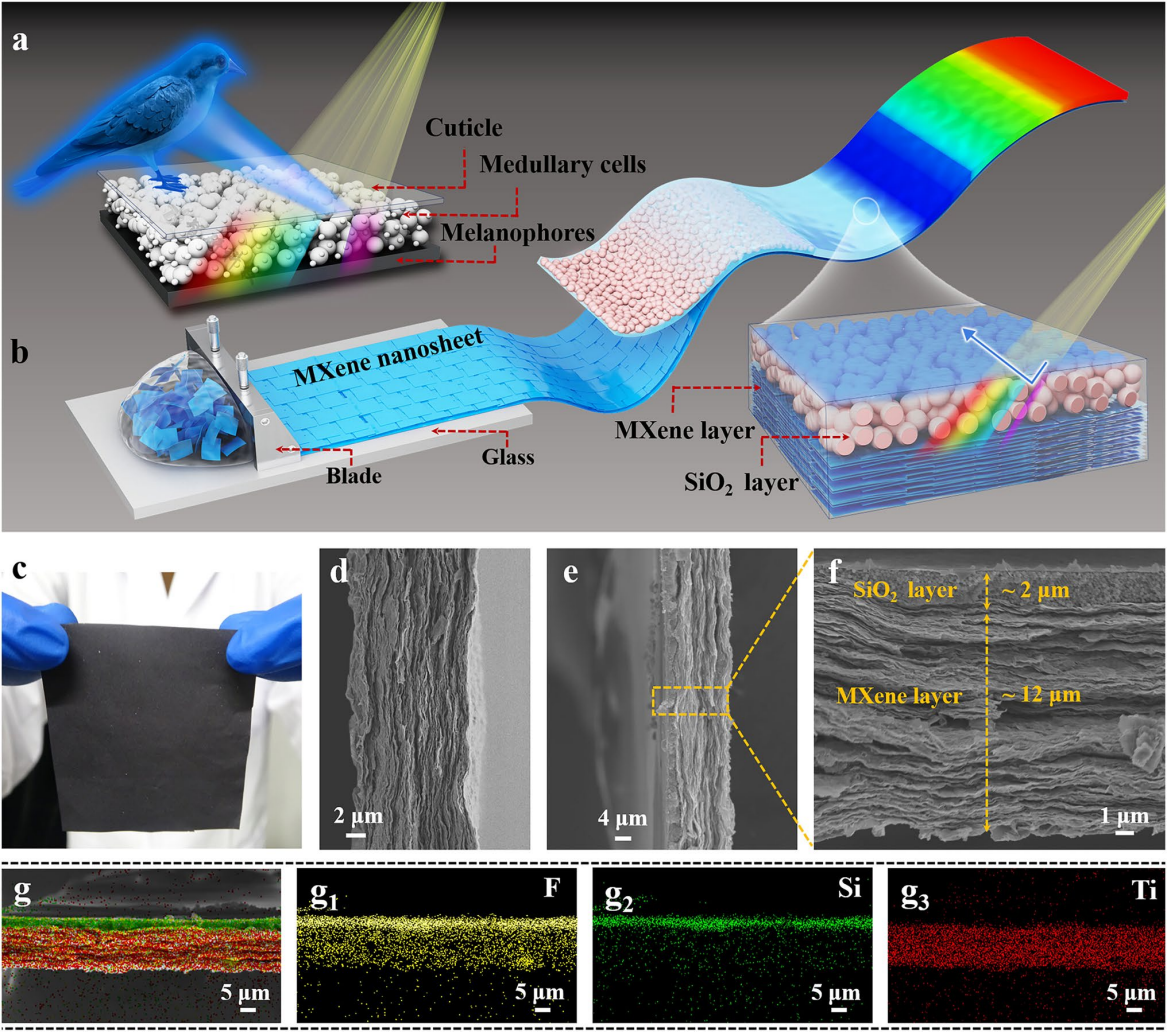
**a** Angle-independent structural color observed in the plumage of the plum-throated cotinga in nature. **b** Schematic fabrication process of MXene-based angle-independent structurally colored soft actuators. **c** Digital photograph of a highly aligned MXene film fabricated via a blade-coating technique and **d** its cross-sectional SEM image. **e** Low-magnification and **f** high-magnification cross-sectional SEM images of biomimetic MXene-based soft actuators and **g** the corresponding elemental mapping images showing the distribution of F, Si, and Ti elements

In general, the angle independence of structural color can be achieved through the development of short-range ordered 3D amorphous photonic nanostructures [26]. In nature, the angle-independent structural color observed in the plumage of the plum-throated cotinga is known to originate from the amorphous arrangement of spongy keratin, isotropically distributed with short-range order within its feather barbs, which is further enhanced by underlying black melanin particles, as shown in Fig. 1a [8, 9]. Recently, artificial short-range ordered 3D amorphous photonic nanostructures loaded with black nanoparticles or enhanced with a black background have been demonstrated to exhibit brilliant angle-independent structural color [27–30]. For example, graphene nanosheets with broad light absorption across the entire visible region were successfully incorporated into monodisperse nanospheres for the fabrication of amorphous photonic nanostructures that show great improvement in color saturation [29]. Cuttlefish ink was used as an additive to produce amorphous photonic structures with high color visibility, which not only break the long-range ordering of the resulting nanostructures but also enhance the color saturation [30]. Bioinspired angle-independent structural color was also developed by depositing oppositely charged materials on the black background substrate and appropriately controlling the thickness of the nanoparticle array [31]. To the best of our knowledge, structurally colored soft actuators with angle independence have not yet been reported.

MXene, known as two-dimensional (2D) transition metal carbides and nitrides, has been in the limelight of research due to the unprecedented combination of multiple promising characteristics, such as broad optical absorption across the UV to infrared regions, excellent hydrophilicity, high thermal conductivity/electrical conductivity and superior photothermal conversion efficiency [32, 33]. Recently, MXene-based smart actuators that can respond to diverse external stimuli, such as electrochemical signals [34, 35], humidity [36, 37], and light [38–43], have been reported. Herein, we report a biomimetic MXene-based soft actuator with brilliant angle-independent structural color, which was fabricated through the self-assembly of colloidal SiO_2_ nanoparticles onto highly aligned MXene films followed by vacuum-assisted infiltration of polyvinylidene fluoride (PVDF) into the interstices (Fig. 1). The formation of the liquid-crystalline phase in colloidal MXene suspensions makes it possible to produce large-scale and highly aligned MXene films using a blade-coating technique [44, 45]. The resulting nanostructured MXene films with a crumpled surface as a black background can not only facilitate the formation of short-range ordered 3D amorphous photonic nanostructures exhibiting angle-independent structural color but also help to significantly improve structural color saturation. Interestingly, the as-prepared angle-independent structurally colored soft actuators were found to exhibit ultrafast actuation and recovery speeds (a maximum curvature of 0.52 mm^−1^ can be achieved within 1.16 s, and a recovery time of ~ 0.24 s) in response to acetone vapor, which can be attributed to the fast acetone absorption/desorption capability of embedded PVDF polymer networks and the resulting anisotropic shape deformation of MXene-based soft actuators. As proof-of-concept illustrations, angle-independent structurally colored soft actuators were used to demonstrate a blue gripper-like bird’s claw, artificial green tendrils, an artificial multicolored butterfly, biomimetic blooming flowers and inchworm-inspired soft walkers, all of which can rapidly respond to acetone vapor. The research disclosed herein can offer new insights into the design and synthesis of advanced photonic nanostructures with angle-independent structural color and pave the way for the development of biomimetic multifunctional soft actuators toward smart soft robotics [46–50].

## Experimental Section

### Materials

Ti_3_AlC_2_ (Carbon-Ukraine), hydrochloric acid (HCl, Sinopharm Chemical Reagents Co., Ltd., Shanghai, China), poly(1,1-difluoroethylene) (PVDF, Sinopharm Chemical Reagents Co., Ltd., Shanghai, China), lithium fluoride (LiF), ammonium hydroxide (NH_3_·H_2_O), tetraethylorthosilicate (TEOS), ethanol, isopropyl alcohol, *N,N*-dimethylformamide (DMF), tetrahydrofuran, dimethylformamide, methanol, dichloromethane, cyclohexane, 2-propanol and acetone were all purchased from Aladdin Chemical Co., Ltd., Shanghai, China.

### Synthesis of Ultrathin MXene Nanosheets

Ti_3_AlC_2_ powder (10 g, < 15 μm particle size) was dispersed in 200 mL of water with 15 cm by magnetic stirring for ~ 10 min. The mixture was left to stand for 8 min to separate small (< 4 μm particle size) Ti_3_AlC_2_ particles. The sedimentation process was repeated three times under the same conditions to fully remove small particles. The collected sediment was dried under vacuum at room temperature for 12 h before being used for synthesizing MXene. 1 g of size-selected Ti_3_AlC_2_ powder was slowly added to the etching solution consisting of 1.6 g lithium fluoride (LiF) in 20 mL of 9 M hydrochloric acid (HCl). Etching was carried out for 36 h at 50 °C. The resulting dispersion was washed with deionized water by repeated centrifugation at 3500 rpm for 5 min per cycle until self-delamination occurred at a supernatant pH of ~ 6. The self-delaminated ultrathin MXene nanosheets were then collected by centrifugation several times at 3500 rpm for 5 min. The dark green supernatant was further centrifuged at 10,000 rpm for 10 min, and the sediment containing MXene nanosheets was collected by drying under vacuum at 60 °C for 12 h.

### Synthesis of SiO_2_ Nanoparticles

The preparation procedure of SiO_2_ nanoparticles was a modified Stöber process [51]. In a typical synthetic procedure of 210 and 260 nm SiO_2_ nanospheres, the total volume of ammonia solution, including deionized water (40 and 28 mL) and ammonium hydroxide (NH_3_·H_2_O, 10 and 22 mL), was 50 mL, to which 100 mL ethanol was then added (Solution A). Another mixture (Solution B), including 18 mL of tetraethylorthosilicate (TEOS) and 82 mL of ethanol, was quickly poured into Solution A (1500 rpm); 2 min later, the stirring rate was decreased to 800 rpm. The preparation process of SiO_2_ nanospheres was finished after 2 h and thoroughly washed with ethanol three times by centrifugation. In a typical synthesis procedure of 315 nm SiO_2_ nanospheres, 0.6 mL of tetraethylorthosilicate TEOS was mixed with 6 mL of NH_3_·H_2_O, 63.3 mL of isopropyl alcohol, and 23.5 mL of deionized water. After stirring for 30 min, 5 mL of TEOS was added dropwise into the above-mixed solution under stirring. The reaction temperature was kept at 35 °C for 2 h. The final solution was carefully collected and washed with distilled water and ethanol three times and dried in a vacuum oven at 60 °C for 12 h. Thus, an 8 wt% SiO_2_ ethanol dispersion stock was prepared for further use.

### Preparation of Nanostructured MXene Films

The MXene slurry exhibiting a liquid crystalline phase was fabricated by mixing N,N-dimethylformamide (DMF) as a solvent (the concentration of MXene in DMF is 28 mg mL^−1^) and 5% PVDF (mass ratio of MXene) as a binder, ground using a mortar, and then dropped onto the surface of a glass slide. Blade-coating MXene films with various thicknesses were prepared using an adjustable blade coating applicator by varying the blade thickness and dried under vacuum at 60 °C for 1 h to evaporate the DMF solvent; The MXene slurry was filter through a cellulose filter membrane (47 mm in diameter, 0.2 μm pore size) to obtained vacuum-filtration MXene films. The surface of the nanostructured MXene film was then treated with oxygen plasma for 10 min to introduce hydroxyl groups for a better combination with the SiO_2_@PVDF layer.

### Fabrication of Angle-Independent Structurally Colored Soft Actuators

The SiO_2_ nanoparticles with the desired size were controllably self-assembled onto the nanostructured MXene film via the dip-coating method (SYDC-100H DIP COATER). The pulling rate was 2 µm s^−1^ at 30 °C, and the dip-coating procedure was carried out three times. The PVDF (6 wt%, in DMF) dispersion was stirred for 5 h at 400 rpm at 80 °C until the solution seemed uniform and transparent. The PVDF dispersion was subsequently infiltrated into the interstices of the resulting MXene/SiO_2_ photonic films in a vacuum oven at room temperature, and the solvent was fully evaporated upon increasing the temperature up to 50, 75, or 100 °C for 30 min. To fabricate a tricolored soft actuator, the colloidal emulsions with different sized SiO_2_ were sequentially self-assembled onto a nanostructured MXene film via the dip-coating method, which was then infiltrated with PVDF dispersion in a vacuum oven at room temperature. Patterned films can be obtained upon the evaporation of the solvent at 75 °C for 30 min and subsequent laser-cutting.

### Characterizations

X-ray diffraction (XRD, Bruker D8 Adv, Germany) was employed to identify the composition and crystal structure of the synthesized samples. Dynamic light scattering (DLS) was conducted on a Malvern zetasizer nanoseries (Nano ZS90) for hydrodynamic particle size determination. FT-IR spectra were collected on a Tensor27 (Bruker, Germany) spectrometer by dispersing completely dried samples in compressed KBr pellets. The wavenumber range was from 400 to 4000 cm^−1^ at a spectral resolution of 2 cm^−1^. The microstructures were observed by field emission scanning electron microscopy (FESEM; S4800, Hitachi, Japan). High-resolution transmission electron microscopy (HRTEM) overview images were obtained on a JEM-2000FX (JEOL, Japan) with an acceleration voltage of 200 kV. HRTEM samples were prepared by dispersing the MXene aqueous solution onto copper grids with the excess solvent evaporated. The measurements of reflection spectra were recorded by a miniature spectrometer (FLAME-S-VIS–NIR-ES, Ocean Insight). The microtopography was investigated under an optical microscope (Nikon, ECLIPSE, LV100N POL).

## Results and Discussion

### Fabrication and Characterizations of Biomimetic MXene-based Soft Actuators

To develop angle-independent structurally colored soft actuators, we first fabricated a highly aligned MXene-based nanostructured film using a blade-coating technique, and the colloidal SiO_2_ nanoparticles were controllably self-assembled onto the crumpled surface of the MXene substrate via a dip-coating method followed by vacuum-assisted infiltration of PVDF into the interstices. Specifically, (1) single-layered MXene nanosheets were synthesized using a modified minimally intensive layer delamination (MILD) method [44, 52]; (2) a repeated sedimentation process was performed to obtain uniformly sized MXene nanosheets exhibiting a lyotropic liquid crystalline phase in *N,N*-dimethylformamide (DMF), whereas large-scale highly aligned nanostructured MXene films with varying thicknesses were facilely prepared through blade-coating colloidal MXene liquid crystals [45, 53]; (3) SiO_2_ nanoparticles with the desired size were controllably self-assembled onto the nanostructured MXene film via a dip-coating method to obtain short-range ordered 3D amorphous photonic nanostructures [54]; (4) a PVDF dispersion was subsequently infiltrated into the interstices of the resulting MXene/SiO_2_ photonic films in a vacuum oven at room temperature, and finally, the solvent was fully evaporated upon increasing the temperature to 75 °C for 30 min. Further details about the synthesis process of MXene are given in the Supporting Information (Figs. S1-S6). Scanning electron microscopy (SEM) was implemented to characterize the nanostructures of the pristine MXene film and the resulting structurally colored soft actuator (MXene/SiO_2_@PVDF). The optical and SEM images of the pristine MXene film are shown in Figs. 1c and S7. A compacted laminar and layer-by-layer nanoarchitecture can be clearly observed on the cross-sectional SEM image (Fig. 1d), and nanostructured wrinkles and wave-like ripples are observed on the surface of the MXene film (Fig. S8). Figure 1e, f show the cross-sectional SEM image of the structurally colored soft actuator with a thickness of ~ 14 μm (SiO_2_ layer ~ 2 μm; MXene layer ~ 12 μm). The energy-dispersive X-ray spectroscopy (EDS) mapping indicates the formation of a bilayered nanostructure composed of a lower MXene layer and an upper SiO_2_ layer, as shown in Fig. 1g. It should be noted that the thickness of different layers can be facilely tailored by simply controlling the blade-coating and dip-coating processes.

### Structural-color Angle-independence of MXene-based Soft Actuators

**Fig. 2:**
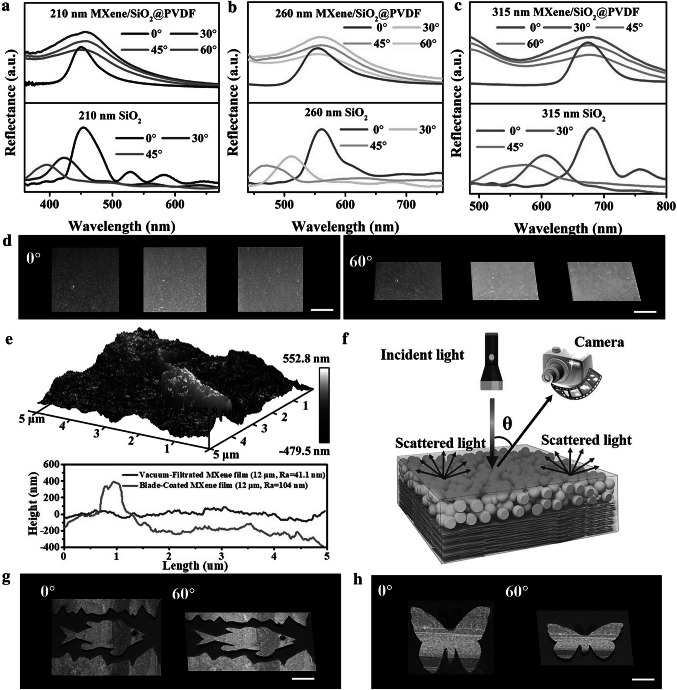
Reflectance spectra of **a** blue, **b** green, and **c** red MXene/SiO_2_@PVDF films (top in figures) and photonic crystals made of pure SiO_2_ nanoparticles (bottom in figures) at varying detection angles relative to the normal direction of the films. **d** Photographs of different MXene/SiO_2_@PVDF films observed at incidence angles of 0° and 60°. **e** AFM images of MXene film prepared via blade-coating method and height profiles of MXene film prepared via the vacuum-filtration and blade-coating methods. **f** Schematic mechanism of the formation of structural color and its angle independence. Photographs of the MXene/SiO_2_@PVDF films with **g** a fish-like pattern and **h** a butterfly-like pattern taken at 0° and 60° incidence angles. Note: The incidence angle is the angle between the incident light and the normal to the point of incidence on a surface; scale bar: 1 cm

The structural-color angle-independence of the MXene-based soft actuators (MXene/SiO_2_@PVDF) was investigated in detail. As shown in the angle-resolved reflection spectra, the wavelength of MXene/SiO_2_@PVDF films barely shifts when the incident angle varies from 0° to 60° (Figs. 2a-c and S9-S15). In contrast, strong angle-dependent reflectance when the incident angle varies from 0° to 60° was observed in the photonic crystals made of pure SiO_2_ nanoparticles (Figs. 2a-c and S16-S19), in which a high long-range order was obtained by directly dip-coating colloidal SiO_2_ nanoparticles on a glass substrate. Photographs of different MXene/SiO_2_@PVDF films further indicate that the appearance of the structural colors remained almost constant at viewing angles from 0° to 60° relative to the normal direction of the films (Figs. 2d and S20). The angle independence of MXene-based structurally colored soft actuators was closely related to the formation of short-range ordered 3D amorphous photonic nanostructures, as shown in the cross-sectional SEM image of the MXene/SiO_2_@PVDF film (Fig. S21). Interestingly, the short-range ordered 3D amorphous photonic nanostructure was closely related to the surface roughness of the MXene film. To better understand the effect of its roughness on structural color, MXene film was prepared via a vacuum-filtration method for comparison. The wrinkles and roughness of the blade-coated MXene film are more pronounced than those of vacuum-filtrated MXene film (Figs. 2e and S22a-f), and as consequence, MXene/SiO_2_@PVDF films prepared using blade-coated MXene film would exhibit evident angle-independence structural color (Fig. S23). Meanwhile, Fig. S22g-i shows that the thickness of blade-coated MXene film has no noticeable influence on its surface nonuniformity, thus the angle-independence structural color is not affected by the thickness of blade-coated MXene film. Physically, the photonic pseudogap, mainly derived from wavelength-specific constructive interference of scattered light due to the short-range order of a certain-sized amorphous array, gives rise to a noniridescent coloration. As schematically shown in Fig. 2f, the broad light absorption of black MXene suppresses wavelength-independent scattering, preventing light transmission from below, prompting the interference of coherent light scattering from amorphous arrays [9, 31, 55]. As a result, the crumpled and highly aligned MXene films can act not only as a supporting layer to facilitate the formation of short-range ordered 3D amorphous photonic nanostructures exhibiting angle-independent structural color but also as a black background to significantly improve structural color saturation. Taking advantage of controllable self-assembly with a dip-coating method, tricolored fish- and butterfly-like patterned films were further demonstrated, in which angle-independent structural colors can still be observed even when changing the viewing angle from 0° to 60° (Figs. 2g, h and S24).

### Actuation Performance of Angle-independent Structurally Colored Soft Actuators

**Fig. 3:**
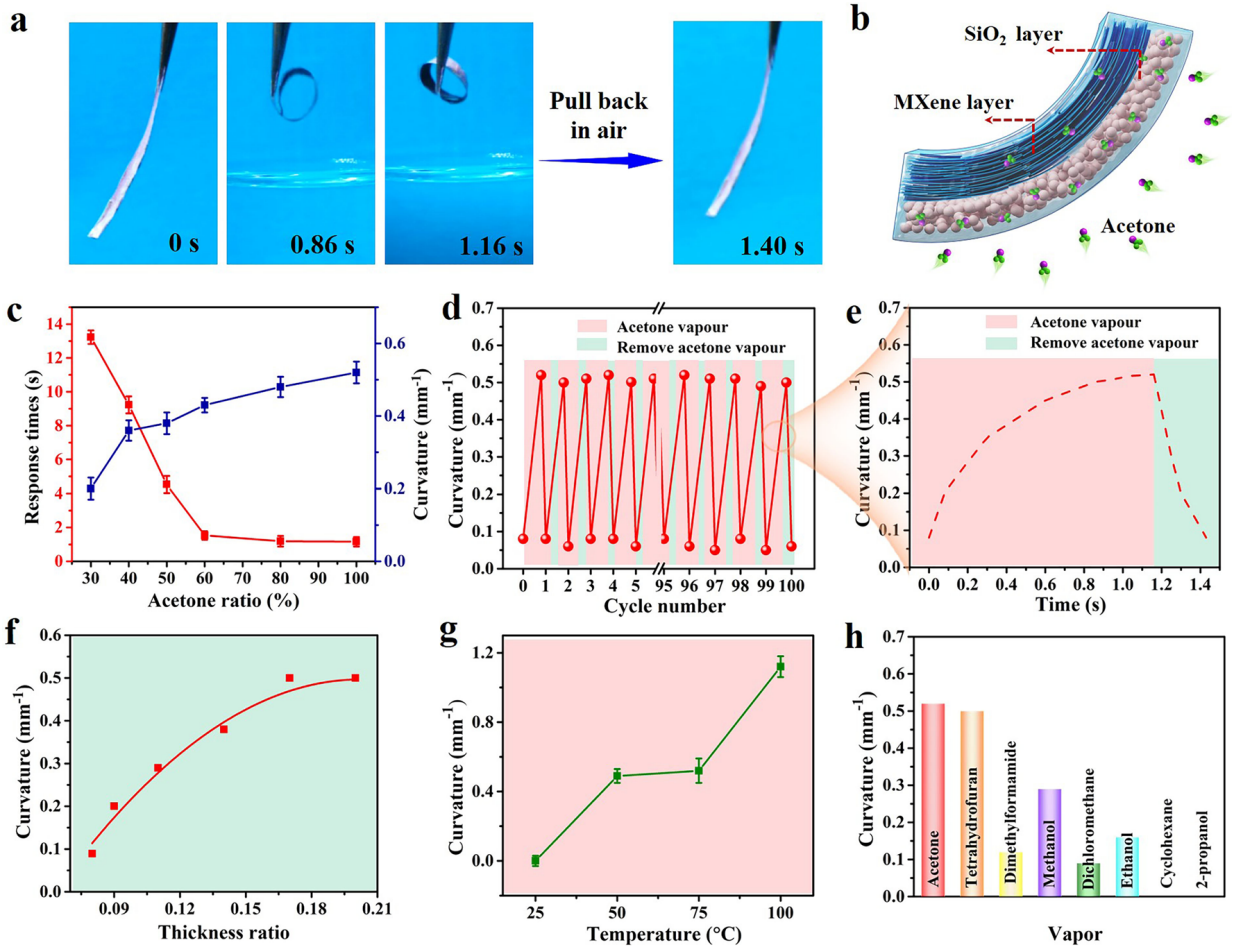
**a** Reversible and ultrafast actuation of the structurally colored soft actuator under exposure to acetone vapor and an air environment. **b** Schematic actuation mechanism of the soft actuator upon exposure to acetone vapor. **c** Dependence of the response time and bending curvature of the soft actuators on the acetone concentration, where the volume ratio of acetone and deionized water increases from 30% (*v/v*) to 100% (*v/v*). **d** The reversibility of the structurally colored soft actuator under cyclic exposure to acetone vapor and an air environment. **e** A complete actuation cycle of the structurally colored soft actuator under exposure to acetone vapor and an air environment. **f** Effect of the thickness ratio of the SiO_2_ layer to the MXene layer on the bending curvature of the soft actuators, where the thickness of the SiO_2_ layer is fixed at ~ 2 μm. **g** Dependence of the bending curvature of the soft actuators on the solvent evaporation temperature. **h** Dependence of the bending curvature of the soft actuator on different organic solvent vapors. Unless otherwise indicated, the size of the MXene/SiO_2_@PVDF film is 20 mm × 2 mm × 14 μm

The actuation performance of structurally colored soft actuators was then investigated under an acetone vapor environment. In the experiment, the structurally colored soft actuator (size: 20 mm × 2 mm × 14 μm, SiO_2_ layer: ~ 2 μm; MXene layer: ~ 12 μm; The *1/r* is the curvature of the structurally colored soft actuator as defined in Fig. S25) was found to exhibit an ultrafast, reversible, and large shape-bending actuation upon cyclic exposure to acetone vapor and an air environment, as shown in Fig. 3a and Movie S1. Such ultrafast actuation outperformed the conventional soft actuators in terms of the response and recovery speed compared with those reported in the literature (Table S1). It should be noted that the structurally colored soft actuators were also found to exhibit excellent mechanical property and a tensile strength of 7 MPa can be obtained (Fig. S26). Figure 3b schematically illustrates the actuation mechanism of the soft actuator upon acetone vapor exposure. The rapid and strong absorption of acetone molecules through the SiO_2_ layer and further their adsorption to the hydrophobic fluorine groups results in a large swelling and volume expansion of the SiO_2_ layer [56, 57], whereas the MXene layer shows low acetone absorption and limited volume change due to its highly aligned and dense topography (Figs. S27-S29), thus yielding an asymmetric shape-bending deformation toward the MXene side. When the soft actuator is moved away from acetone vapors, it immediately releases the solvent by evaporation and recovers its original shape. Figure 3c indicates that the response speed and maximum shape-bending curvature of the structurally colored soft actuator can be easily modulated by changing the volume ratio of acetone in deionized water. The acetone vapor-driven actuation process is fully reversible and reproducible for many actuation cycles without noticeable degradation (Fig. 3d, e). Interestingly, the maximum shape-bending curvature, response speed, and recovery speed of the structurally colored soft actuator were found to significantly depend on the thickness ratio of the active SiO_2_ layer and passive MXene layer. When the thickness of the SiO_2_ layer is fixed at ~ 2 μm, the maximum shape-bending curvature increases with increasing thickness ratio, as shown in Fig. 3f, and similarly, a decrease in the thickness of the MXene layer increases the shape-bending and recovery speed (Fig. S30). Moreover, it was found that the vapomechanical actuation behaviors of the soft actuators could also be influenced by the temperature of solvent evaporation in the fabrication process, as well as by the polarity of the vapors in the actuation process (Figs. 3g, h and S31-S34).

**Fig. 4:**
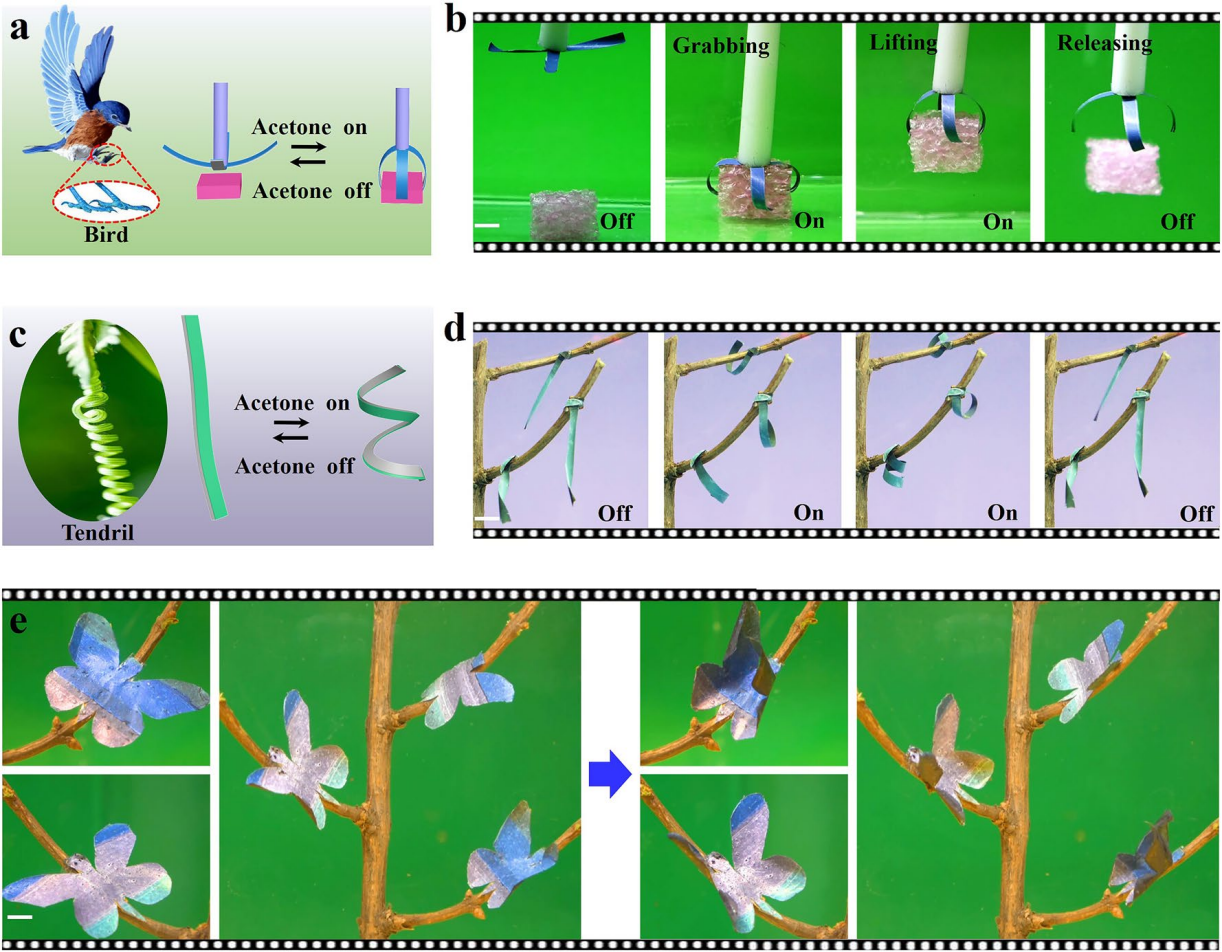
**a, b** A blue gripper with angle-independent structural color that can capture the target inspired by the bird’s claw. **c, d** Artificial tendrils with angle-independent green structural color that can reversibly twine around the tree branches. **e** Artificial butterflies with multiple angle-independent structural colors whose wings flutter up and down on tree branches upon cyclic exposure to acetone vapor (Scale bar: 0.5 cm)

As a proof-of-concept illustration, the angle-independent structurally colored soft actuator was applied for fabricating a blue gripper-like bird’s claw that can capture the target (Fig. 4a). As shown in Fig. 4b and Movie S2, the soft gripper with angle-independent blue structural color can controllably grasp, lift up and release an object upon cyclic exposure to acetone vapor. Inspired by the tendrils of climber plants, artificial tendrils with angle-independent green structural color were developed to reversibly twine around the tree branches upon cyclic exposure to acetone vapor (Fig. 4c, d and Movie S3). Interestingly, artificial butterflies with angle-independent multiple structural colors were further devised with an as-prepared MXene-based soft actuator, where the wings of the butterfly can dynamically flutter up and down on tree branches upon cyclic exposure to acetone vapor (Fig. 4e and Movie S4). Moreover, angle-independent structurally colored soft actuators were also used to demonstrate a biomimetic flower that shows a dynamic blooming behavior upon acetone vapor exposure (Fig. S35) and an inchworm-inspired soft walker that can propel itself spontaneously on a ratcheted substrate under cyclic acetone vapor exposure (Fig. S36).

## Conclusions

In this work, we report the design and fabrication of MXene-based structurally colored soft actuators with angle independence toward biomimetic soft robots. The integration of both angle-independent structural color and actuation functions in a single monolithic material system was enabled by self-assembly of colloidal SiO_2_ nanoparticles onto highly aligned MXene films followed by vacuum-assisted infiltration of PVDF into the interstices. The introduction of MXene nanomaterials into structurally colored soft actuators is advantageous in many aspects: (1) Colloidal MXene liquid crystals facilitate the fabrication of large-scale highly aligned nanostructured MXene films with varying thicknesses via a blade-coating method; (2) The resulting nanostructured MXene films with a crumpled surface can not only facilitate the formation of short-range ordered 3D amorphous photonic nanostructures exhibiting angle-independent structural color but also help to significantly improve structural color saturation; (3) The MXene nanostructured film as a passive layer of soft actuator shows low acetone absorption and limited volume change due to its highly aligned and dense topography, which results in a large swelling and volume expansion of the SiO_2_ layer and an asymmetric shape-bending deformation toward the MXene side. Interestingly, the resulting MXene/SiO_2_@PVDF film was found to exhibit constant structural colors at viewing angles from 0° to 60° relative to the normal direction of the films, which can be attributed to the formation of short-range ordered 3D amorphous photonic nanostructures on the surface-crumpled MXene film. Importantly, the structurally colored soft actuators exhibit ultrafast, reversible, and large shape-bending actuation upon cyclic exposure to acetone vapor and air (a maximum curvature of 0.52 mm^−1^ is observed at 1.16 s) and recovery (~ 0.24 s) due to the anisotropic nanostructures of MXene-based soft actuators and the fast acetone insertion/extraction capability of embedded PVDF polymer networks. It should be noted that the vapomechanical actuation behaviors of the structurally colored soft actuators could be significantly influenced by diverse factors, such as the volume ratio of acetone in deionized water, the thickness ratio of the active SiO_2_ layer and passive MXene layer, and the polarity of the vapors in the actuation process. As proof-of-concept illustrations, angle-independent structurally colored soft actuators were used to demonstrate a blue gripper such as bird’s claw that can capture the target, artificial green tendrils that can twine around the tree branches, and artificial multicolored butterflies that can flutter the wings, as well as biomimetic blooming flowers and inchworm-inspired soft walkers, upon cyclic exposure to acetone vapor. This research is expected to shed new light on the design and synthesis of advanced photonic nanostructures with angle-independent structural color and provide useful insights for the development of biomimetic multifunctional soft actuators toward somatosensory soft robotics, next-generation intelligent machines and thermal regulation materials [58–63].

### Supplementary Information

Below is the link to the electronic supplementary material.

Supplementary file1 (MP4 3660 KB)

Supplementary file2 (MP4 7954 KB)

Supplementary file3 (MP4 5023 KB)

Supplementary file4 (MP4 5386 KB)

Supplementary file5 (PDF 1487 KB)
