# Supplementary material for: Bioinspired MXene-Based Soft Actuators Exhibiting Angle-Independent Structural Color
Source: Nanomicro Lett. 2022 Nov 28;15:1. doi: 10.1007/s40820-022-00977-4 (PMC9705670; doi:10.1007/s40820-022-00977-4)
Supplement: Supplementary file 5 — Supplementary file5 (PDF 1487 KB) [file 40820_2022_977_MOESM5_ESM.pdf]

Supporting Information for

Bioinspired MXene-Based Soft Actuators Exhibiting Angle-Independent Structural Color

Pan Xue[1], Yuanhao Chen[1], Yiyi Xu[2], Cristian Valenzuela[1], Xuan Zhang[1], Hari Krishna Bisoyi[3], Xiao Yang[1], Ling Wang[1, *], Xinhua Xu[1*], and Quan Li[2, 3, *]

[1] School of Materials Science and Engineering, Tianjin University, Tianjin 300350, P. R. China

[2] Institute of Advanced Materials, School of Chemistry and Chemical Engineering, and Jiangsu Province Hi-Tech Key Laboratory for Biomedical Research, Southeast University, Nanjing 211189, P. R. China

[3]Advanced Materials and Liquid Crystal Institute and Chemical Physics Interdisciplinary Program, Kent State University, Kent, Ohio 44242, USA

*Corresponding authors. E-mail: lwang17@tju.edu.cn (L. W.); xhxu@tju.edu.cn (X. X.); quanli3273@gmail.com (Q. L.)

S1 Fabrication and Characterizations of MXene-based Soft Actuators

We synthesized MXene using LiF-HCl etching of Ti_3AlC_2 MAX phase by preselecting large Ti_3AlC_2 MAX phase particles from the as-received MAX phase prior to the selective etching step (**Fig. S1**), also referred to as the modified minimally intensive layer delamination (MILD) method, as illustrated in **Fig. S2**. All the X-ray diffraction (XRD) peaks can be well indexed to $Ti_3C_2T_x$ (red line), which demonstrates that Ti_3AlC_2 (JCPDS No. 52-0875, blue line) was successfully etched of Al layers (**Fig. S3**). The thin and transparent flakes of exfoliated $Ti_3C_2T_x$ nanosheets can be clearly seen in transmission electron microscopy (TEM) image, showing the typical 2D sheet-like structure (**Fig. S4a**). The high-resolution images show that the constant interplanar distance is ~0.13 nm and MXene exhibits a hexagonal crystalline nature, as shown by the selected area electron diffraction (SAED) pattern in **Fig. S4b**. These results are implemented to structural characterization of ultrathin MXene, indicating a successful formation of single-layer MXene nanosheets using a modified minimally intensive layer delamination (MILD) method. In addition, the dynamic light scattering characterization shows that the average hydrodynamic diameters of MXene nanosheets in N,N-dimethylformamide (DMF) are ~1 μm (**Fig. S5**). We also demonstrated that the lyotropic liquid crystalline phase of MXene nanosheets can be achieved using a solvent compatible with MXene, including deionized water and *N*,*N*-dimethylformamide (DMF) (**Fig. S6**).

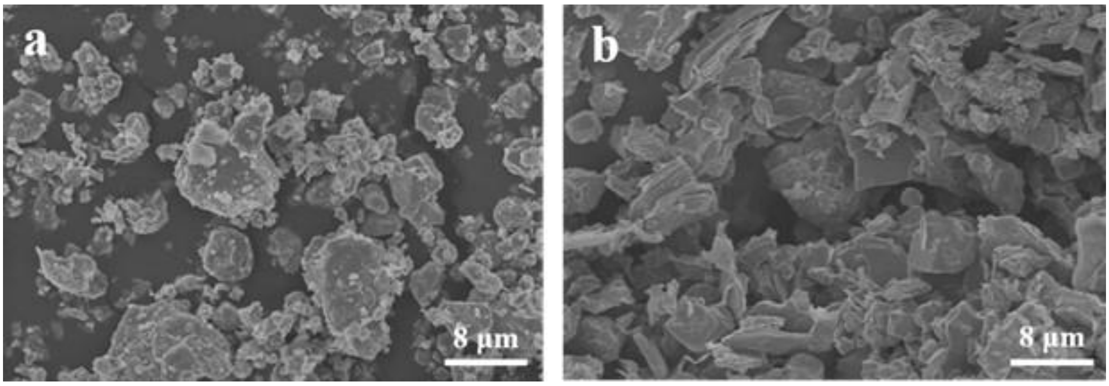

Fig. S1 SEM images of Ti_3AlC_2 (**a**) before and (**b**) after size selection

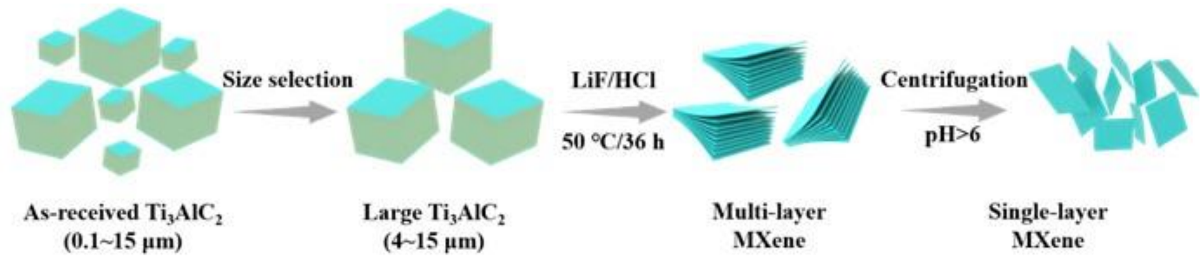

Fig. S2 Schematic process of modified minimally intensive layer delamination (MILD) method

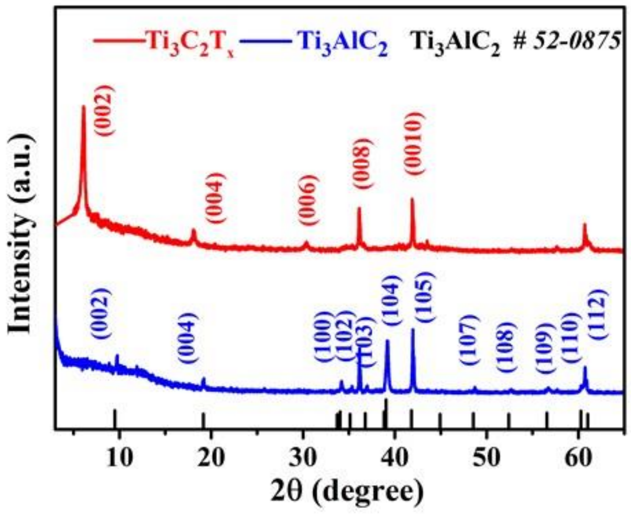

Fig. S3 XRD patterns of the Ti_3AlC_2 and MXene

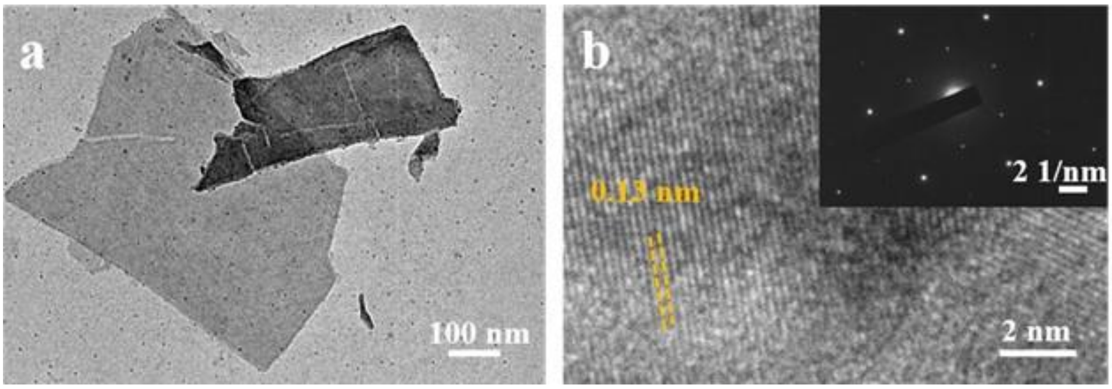

Fig. S4 (**a**) TEM image of the single-layer MXene. (**b**) Magnified TEM image of the single-layer MXene, and the corresponding SAED pattern in the inset

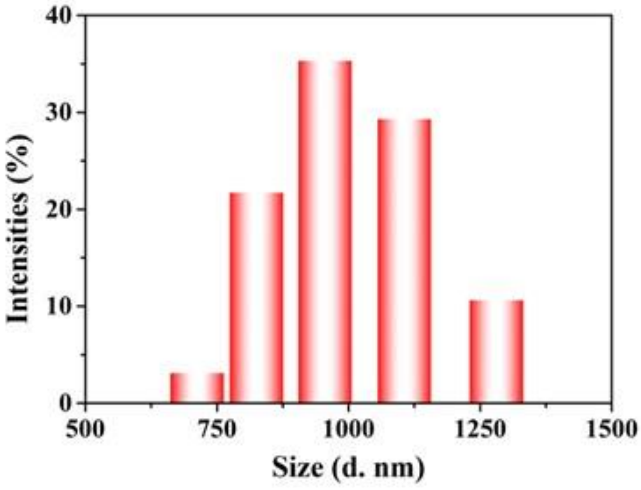

Fig. S5 Particle-size distribution of MXene dispersions in N,N-dimethylformamide (DMF)

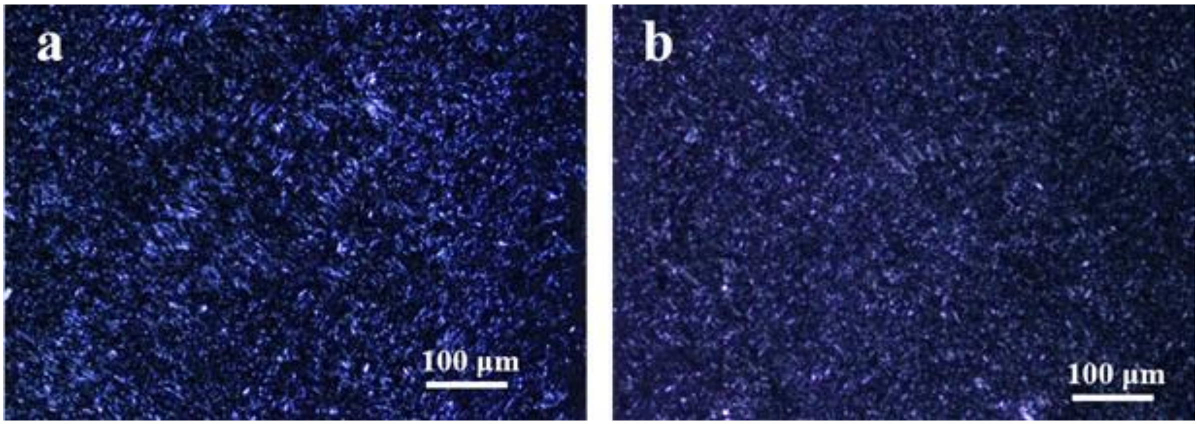

Fig. S6 Characterizations of colloidal MXene liquid crystals: POM images of 28 mg/mL MXene dispersions in (**a**) deionized water, (**b**) *N*,*N*-dimethylformamide (DMF)

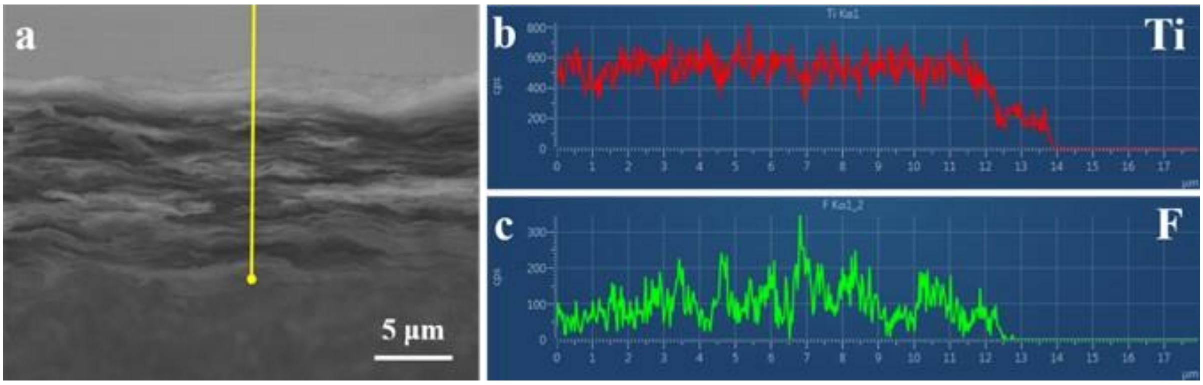

Fig. S7 SEM image and EDX spectra of the MXene film

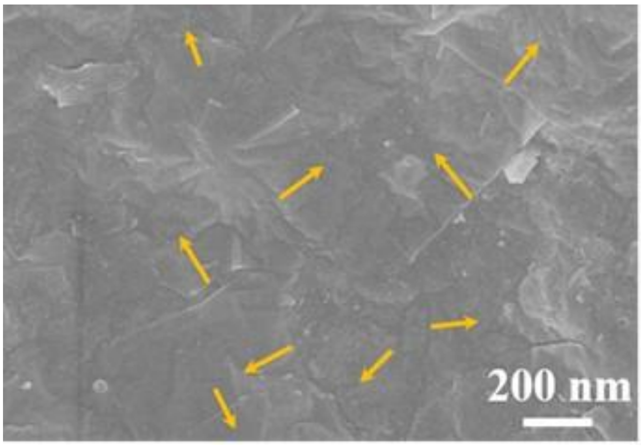

Fig. S8 SEM image of the surface of the MXene film

S2 Optical Properties of Angle-Independent Structural Color Soft Actuators

The preparation procedure of SiO_2 nanospheres was a modified Stöber process. TEM, SEM images taken from both the interior and the surface of samples show that the whole structure is quite homogeneous and the average diameters of SiO_2 nanospheres are ~210, 260, and 315 nm (**Figs. S9 and S10**). In addition, the dynamic light scattering (DLS) characterization shows that the average hydrodynamic diameters of SiO_2 nanospheres in deionized water are ~236 nm, 289 nm, 340 nm (**Fig. S11**). Hydrodynamic sizes of these microspheres measured by nanoparticle size measurement are bigger than the actual measured diameters from the SEM and TEM. DLS exhibits a hydrodynamic size that corresponds to the core and the swollen corona of the micelles, whereas SEM and TEM often exhibit the size of the core for micelles in a dried state as the corona with low electronic density is not visible [S1].

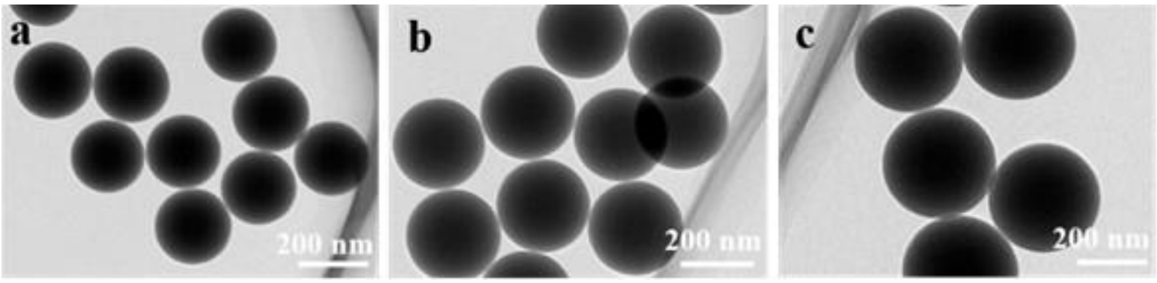

Fig. S9 TEM images of different SiO_2 nanospheres: (**a**) 210 nm, (**b**) 260 nm, (**c**) 315 nm

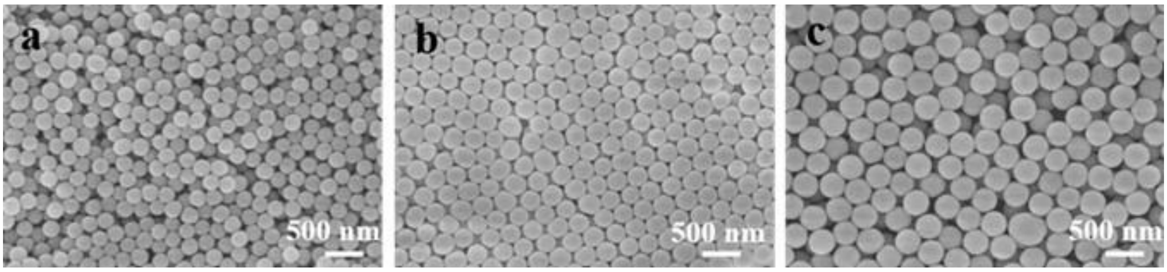

Fig. S10 SEM images of different SiO_2 nanospheres: (**a**) 210 nm, (**b**) 260 nm, (**c**) 315 nm

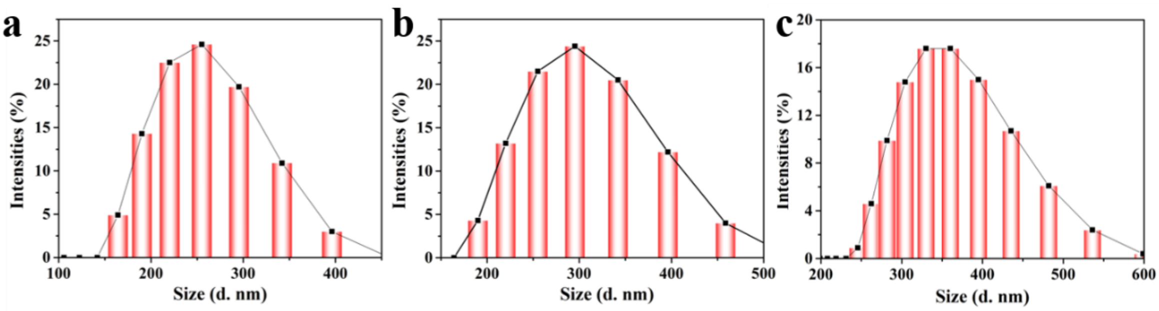

Fig. S11 Particle-size distribution of different SiO_2 nanospheres: (**a**) 210 nm, (**b**) 260 nm, (**c**) 315 nm dispersions in ethyl alcohol

The MXene/SiO_2@PVDF films with SiO_2 nanospheres of 210, 260, and 315 nm in diameter showed reflection peaks at 445, 550, and 662 nm, respectively (**Fig. S12**). The measured reflection spectra of these films were converted into the more standardized Commission Internationale de L'Eclairage (CIE) chromaticity value, which verified that the blue, green, red-structural color films were prepared (**Fig. S13**). **Figure S14** presents digital images of three different colored films, blue, green and red, which are composed of SiO_2 nanospheres with diameters of 210, 260, and 315 nm, respectively. **Figure S15** shows the barely changes of the reflection spectra peak positions of the three colored MXene/SiO_2@PVDF film as the incidence angles increased from 0° to 60°.

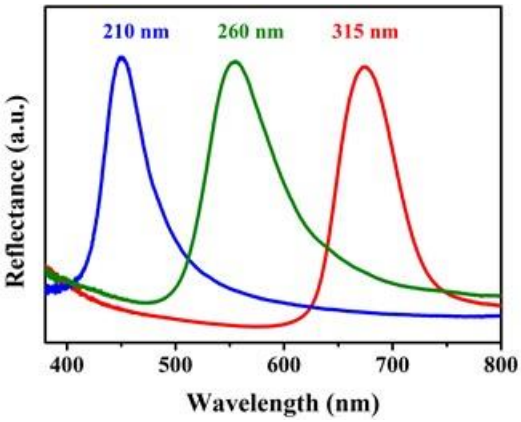

Fig. S12 The reflection spectra of the three colored MXene/SiO_2@PVDF films with 210, 260, and 315 nm diameter sizes

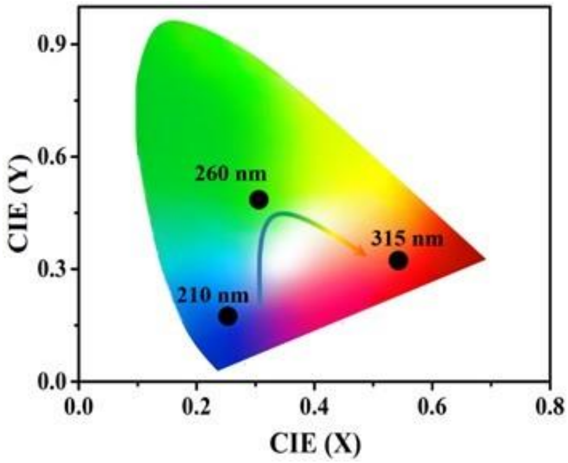

Fig. S13 Positions of the three colored MXene/SiO_2@PVDF films in the Commission Internationale de L'Eclairage (CIE) chromaticity diagram

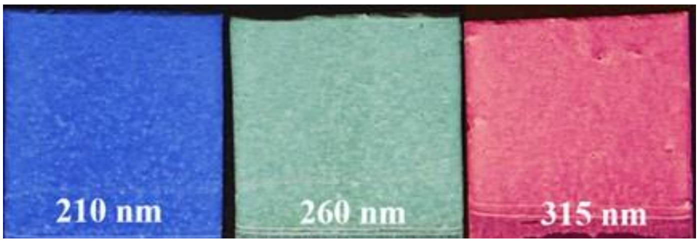

Fig. S14 Digital photographs of the three colored MXene/SiO_2@PVDF films, blue (210 nm SiO_2 nanospheres), green (260 nm SiO_2 nanospheres), and red (315 nm SiO_2 nanospheres)

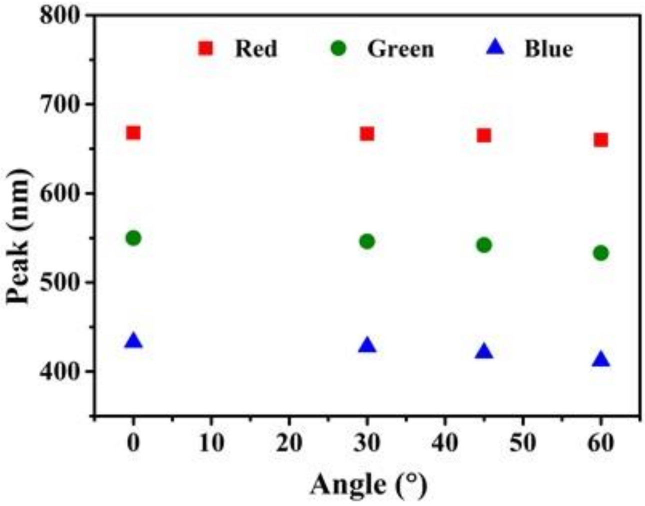

Fig. S15 Changes of the reflection spectra peak positions of the three colored MXene/SiO_2@PVDF films as the incidence angles increased from 0° to 60°

In contrast, the 3D photonic crystals with only SiO_2 nanoparticles, in which high long-range order was obtained by directly dip-coating colloidal SiO_2 nanoparticles on the glass substrate, are named SiO_2 photonic crystal films. The SiO_2 nanospheres were assembled into highly ordered SiO_2 photonic crystal films, as marked by a red hexagon (FCC ordered arrays), which can be observed on the top-surface and cross-sectional scanning electron microscopy (SEM) images in **Figs. S16 and S17**. The highest reflection peak of the SiO_2 photonic crystal films was achieved when the dip-coating was three times, whereas the effect of the SiO_2 photonic crystal films thickness on the reflection spectra is also illustrated in **Fig. S17**. The digital photos of the SiO_2 photonic crystal films (210, 260, and 315 nm) exhibit different colors of blue, green, and red at a vertical angle (**Fig. S18**). Meanwhile, the SiO_2 photonic crystal films of diameters 210, 260, and 315 nm showed reflection peaks at 445, 550, and 662 nm, respectively (**Fig. S19**).

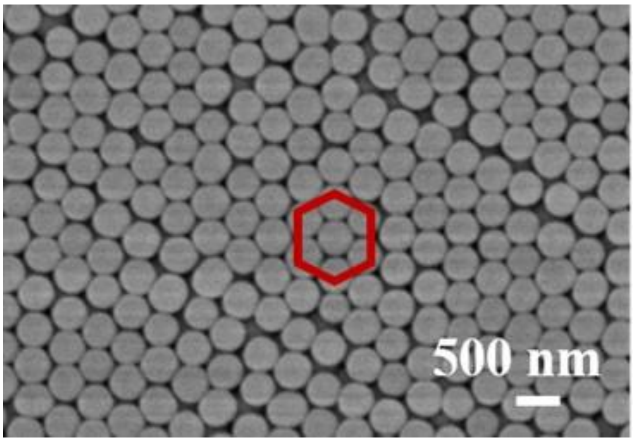

Fig. S16 SEM image of the SiO_2 photonic crystal films

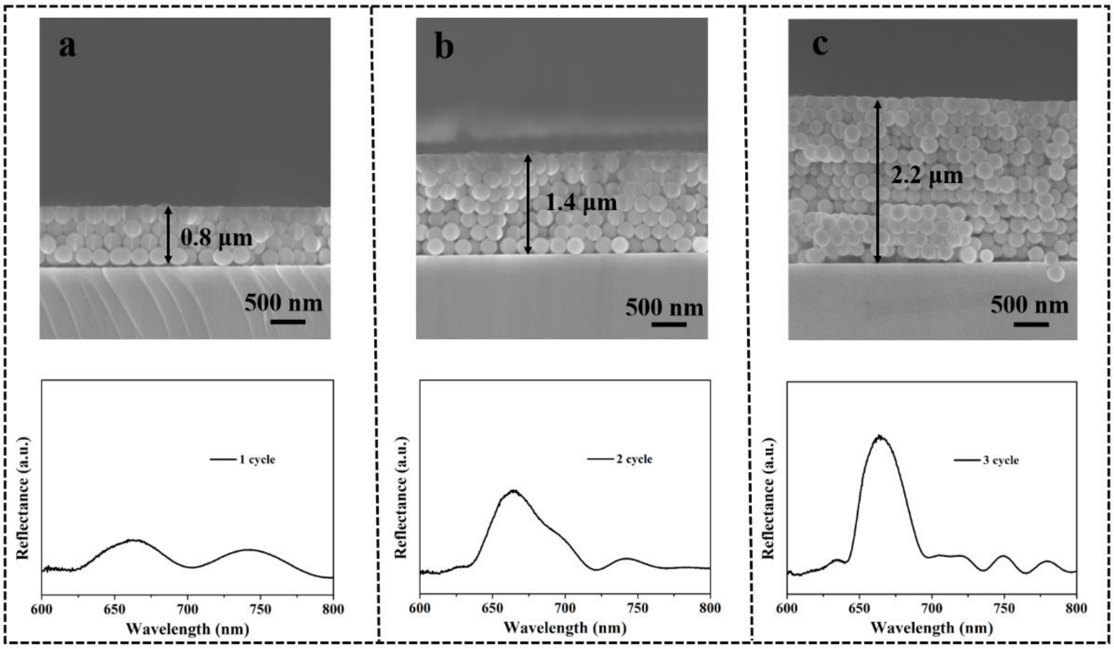

Fig. S17 The SiO_2 photonic crystal films. The SiO_2 photonic crystal films assembled by dip-coating method for (**a**) one, (**b**) two, and (**c**) three times coating (top) and the corresponding reflection spectra (down). 315 nm SiO_2 was used in this figure

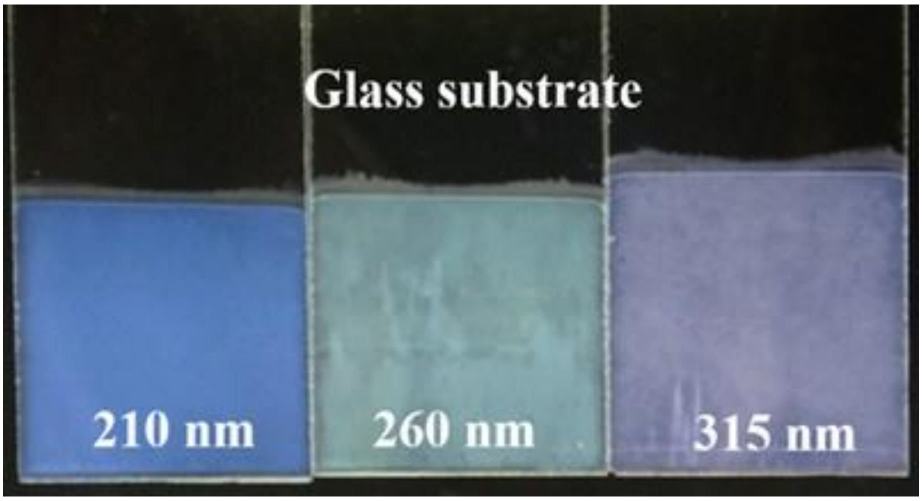

Fig. S18 SiO_2 photonic crystal films assembled by three times dip-coating method on the glass substrate with 210, 260, and 315 nm diameter size

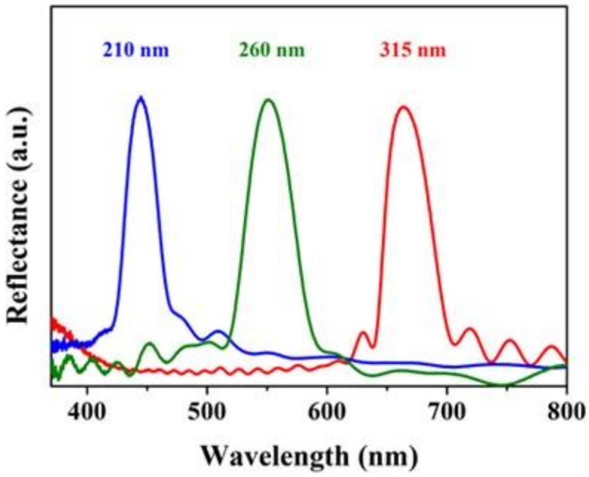

Fig. S19 The reflection spectra of SiO_2 photonic crystal films on the glass substrate with 210 nm, 260 nm, and 315 nm diameter sizes

Photographs of different MXene/SiO_2@PVDF films further indicate the appearance of the structural colors remained almost constant at viewing angles from 0° to 60° relative to the normal direction of the films (**Fig. S20**). The angle-independence of MXene-based structural color soft actuators was closely related to the formation of short-range ordered 3D amorphous photonic nanostructures, as shown in the cross-sectional TEM image of MXene/SiO_2@PVDF film (**Fig. S21**). Taking advantage of controllable self-assembly with a dip-coating method, a MXene/SiO_2@PVDF film with color gradient was prepared by regional dip-coating method. Firstly, a piece of glass slide was put in emulsions A, and dip-coated a certain height. The part which was immersed in emulsion A is cleaned up, and then the glass was put in emulsion B for the dip-coating process. Repeat the above steps for emulsion C. Finally, a tricolor template is obtained, which was made up of SiO_2 nanospheres with various diameters (**Fig. S24**).

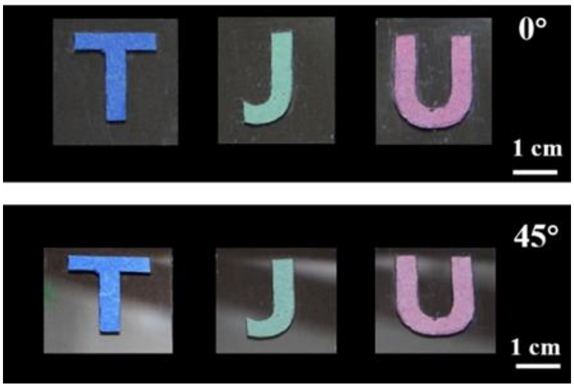

Fig. S20 Photographs of the MXene/SiO_2@PVDF films with "TJU" pattern taken at 0° and 45° incidence angles

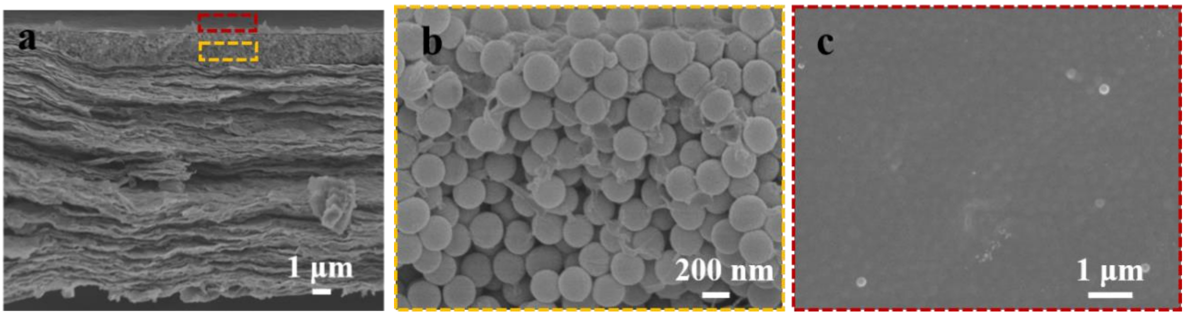

Fig. S21 Morphologies and characterizations of the MXene/SiO_2@PVDF films. (**a**) The cross-sectional SEM image of MXene/SiO_2@PVDF film. (**b**)The high-magnification cross-sectional SEM image of the SiO_2 layer. (**c**) The top-viewed SEM image of the SiO_2 layer

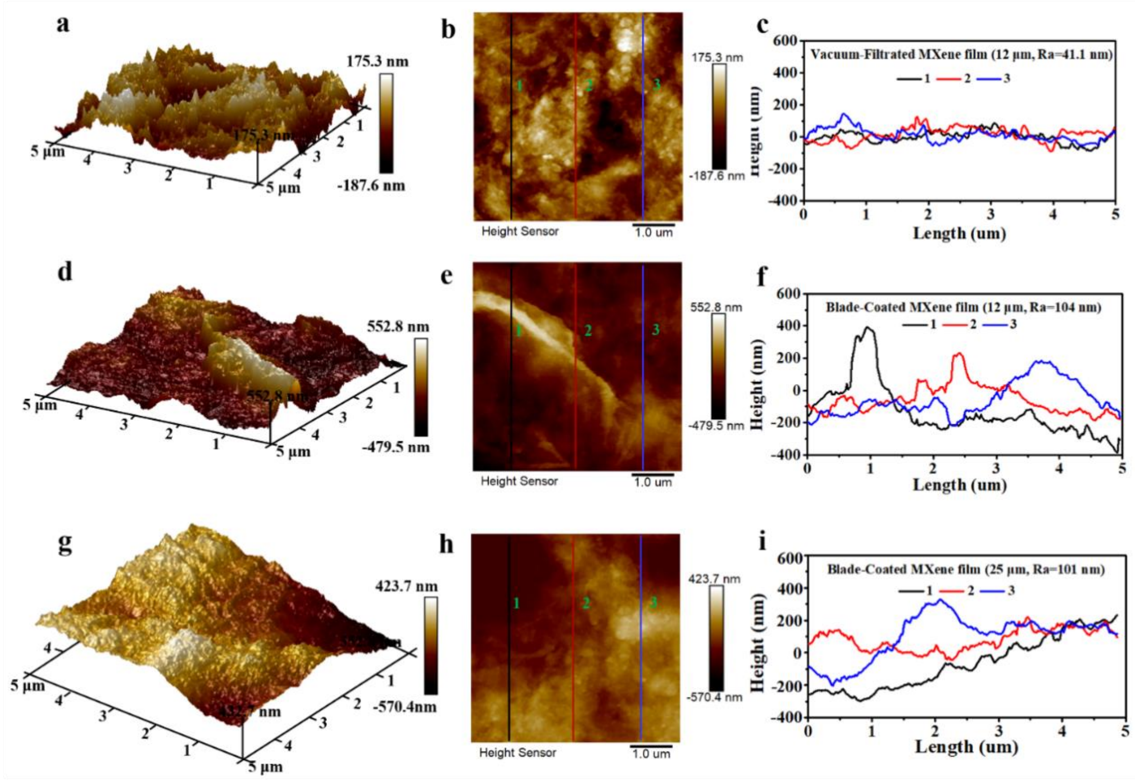

Fig. S22 3D and 2D AFM images of MXene film and height profiles. (**a-c**) 12 μm MXene film prepared *via* the vacuum-filtration method. (**d-f**) 12 μm MXene film prepared *via* the blade-coating method. (**g-i**) 25 μm MXene film prepared *via* the blade-coating method

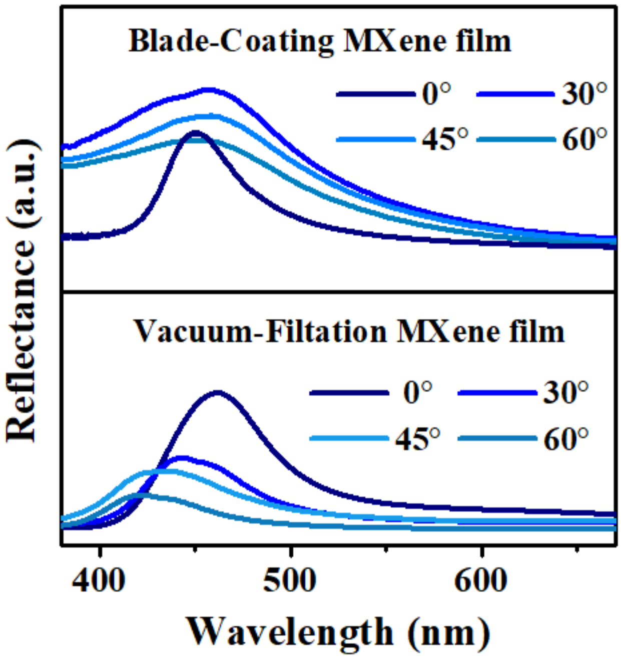

Fig. S23 Reflection spectra of MXene/SiO_2@PVDF films made by blade-coating MXene film and vacuum-filtration MXene film

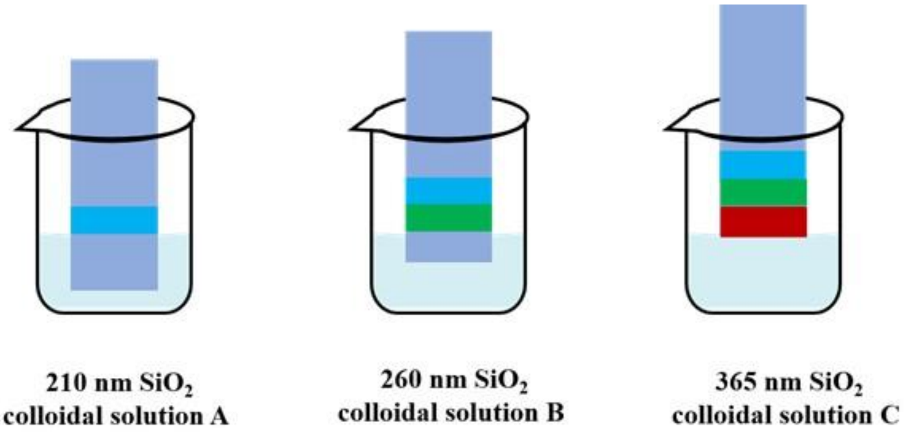

Fig. S24 Schematic diagram of the regional dip-coating method

S3 Actuation Performance of Angle-Independent Structural Color Soft Actuators

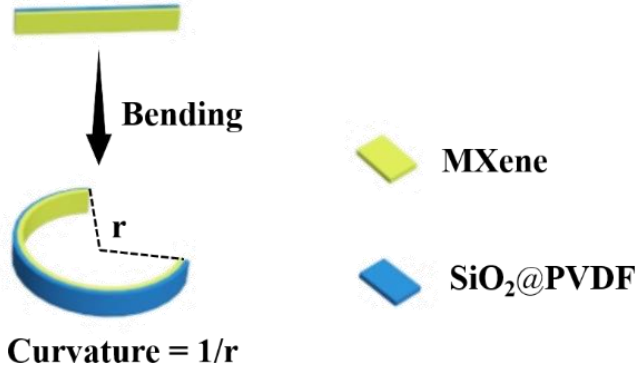

Fig. S25 Schematic illustration of bending angle (curvature) in acetone vapor-driven structurally colored soft actuators. First, the structurally colored soft actuators are cut into strip (20 mm × 2 mm × 14 μm). Second, the strip is attached to a pair of pincers. Then, the actuation is tested when the structurally colored soft actuators exposure to acetone vapor and an air environment. Finally, the 1/r is the curvature of the structurally colored soft actuators to represent shape-bending angle

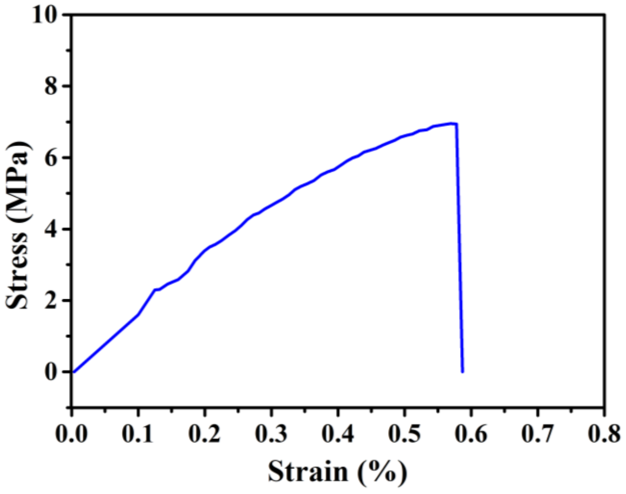

Fig. S26 Strain-stress curve of the MXene/SiO_2@PVDF film

The actuation performance of structural color soft actuators was then investigated under an acetone vapor environment. After exposure of SiO_2@PVDF film to acetone vapor, a typical peak signal came out at 1708 cm^{-1}, which was enhanced with prolonging acetone vapor-

exposure time, indicating the adsorption of acetone molecules into PVDF structure (**Fig. S27**). **Figure S28** show the FT-IR spectra for monitoring the acetone vapor absorption of MXene film. The profiles of MXene film have no visible absorbance change at 1708 cm^{-1} upon prolonging acetone vapor-exposure time. It is indicated that the MXene film shows low acetone adsorption due to its highly aligned and dense topography. In addition, a microbalance (Mettler Toledo) was utilized to monitor the mass variation of the films (**Fig. S29**). For the SiO_2@PVDF film, the initial weight was 17.55 mg which was defined as W_0. After absorption of acetone vapor for 10 s, the film's weight was recorded and marked as W_a. The film was then allowed to desorb acetone molecules in air for 10 s, and the weight was recorded and defined as W_b. Total five cycles were repeated. Acetone vapor sorption ratio in each cycle was calculated as: $(W_a - W_0) \times 100\% / W_0$ and $(W_b - W_0) \times 100\% / W_0$ respectively. In the case of MXene film, the data was obtained as the same calculating method, and the initial weight of MXene was 15.91 mg. The results indicate that SiO_2@PVDF film is capable of adsorption of acetone vapors, and desorbing them rapidly, but MXene film doesn't show significant mass change with acetone adsorption and desorption. Thus, the actuation mechanism of MXene/SiO_2@PVDF film is driven by the rapid and strong adsorption of acetone molecules in the hydrophobic fluorine groups of SiO_2@PVDF film, whereas the MXene layer shows low acetone adsorption and limited volume change due to its highly aligned and dense topography, thus yielding an asymmetric shape-bending deformation towards the MXene side.

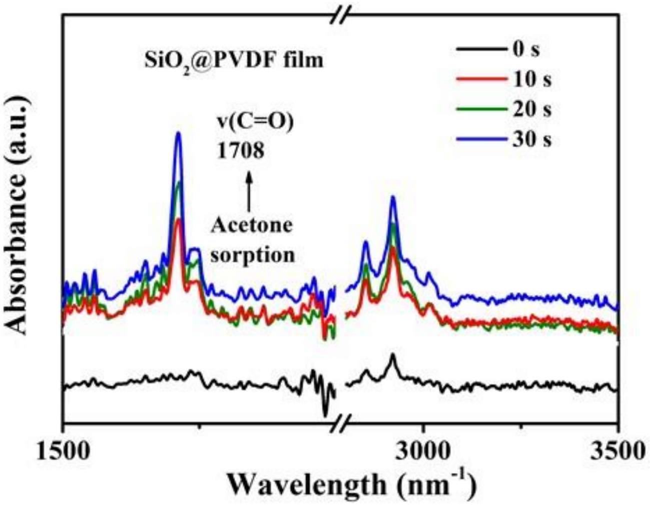

Fig. S27 FT-IR spectra for monitoring the acetone vapor absorption of SiO_2@PVDF film

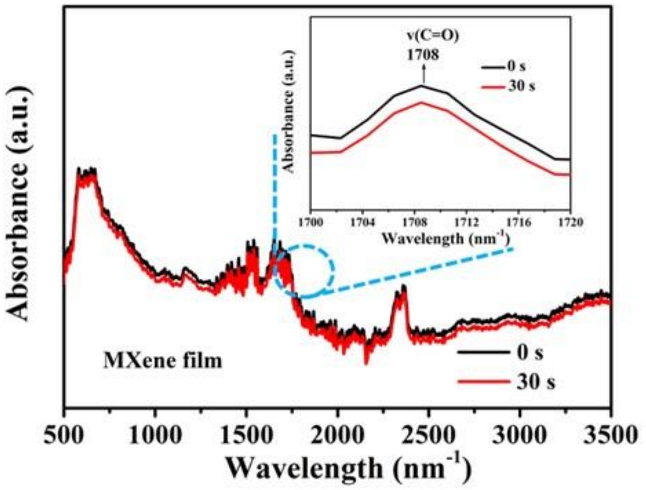

Fig. S28 FT-IR spectra for monitoring the acetone vapor absorption of MXene film

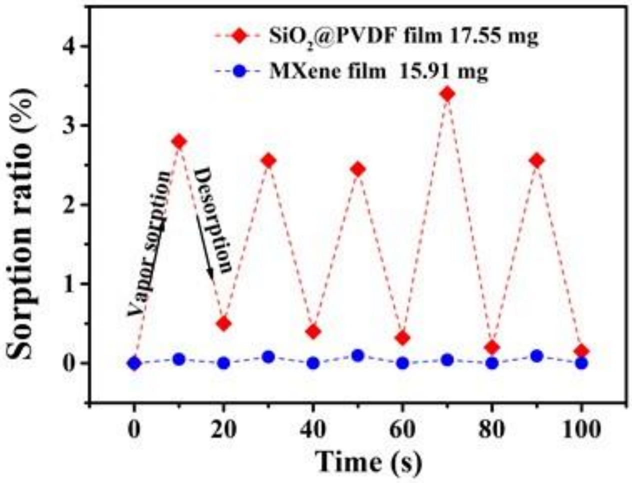

Fig. S29 Acetone-vapor absorption performance of SiO_2@PVDF and MXene film

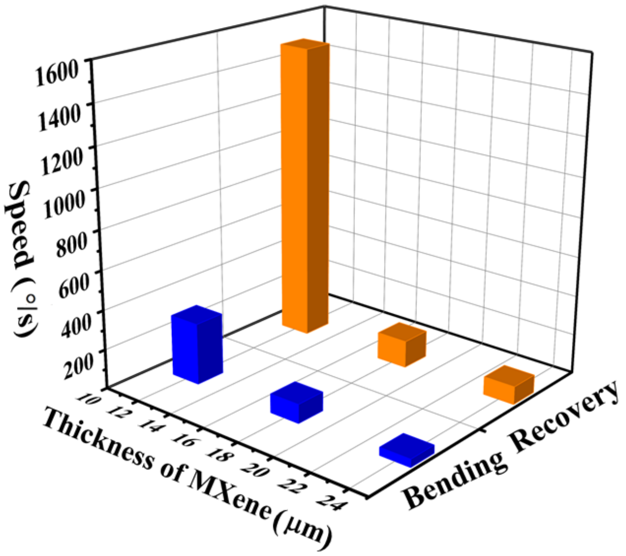

Fig. S30 Relationship between the thickness of MXene layer and bending/recovery speed

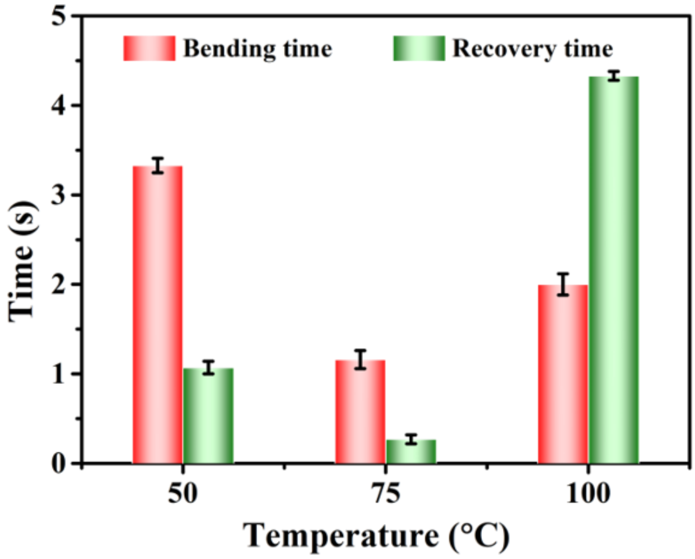

Fig. S31 Relationship between the solvent evaporation temperature of PVDF solution during the fabrication process and bending/recovery time

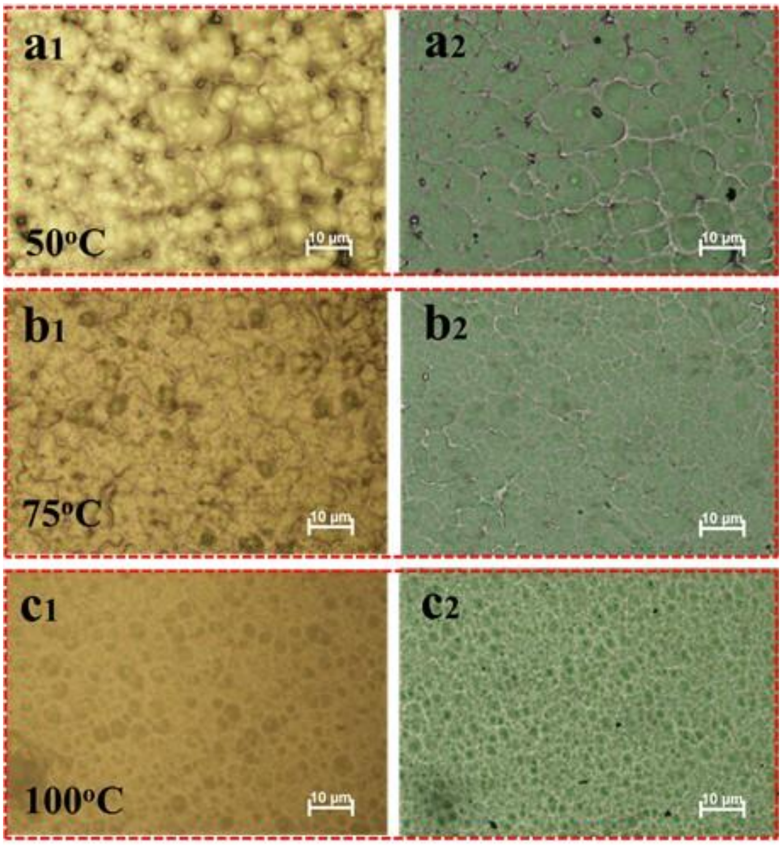

Fig. S32 Optical microscope images of PVDF film deposited at different temperatures of solvent evaporation. (**a**) 50 °C, (**b**) 75 °C, and (**c**) 100 °C. (**a1-c1**) Using optical microscope images of the reflected light, (**a2-c2**) Using optical microscope images of the transmitted light.

Optical microscope images of PVDF film deposited at different temperatures of solvent evaporation: 50, 75, and100 °C. The diameter of formed microspheres at 100 °C reaches ~3 μm that results in a surface pore size much smaller and denser than that of the PVDF film prepared at 50 and 75 °C. Too small and dense pore size actually is favorable to acetone absorption but not favorable to facile desorption of acetone. Therefore, the soft actuators prepared at 100 °C of PVDF solvent evaporation could have the maximum bending curvature and slowly recovery speed

S4 Biomimetic Proof-of-concept Illustrations Based on Angle-independent Structural Color Soft Actuators

Upon exposure to acetone vapor, the swelling of the active layer causes downward bending of the initially curved, angle-independent structural color soft actuator. At this point, because the ratcheted substrate surface prevents backward sliding, the back edge bearing a higher frictional force ($F2 > F1$) acts as a stationary end, leading to the front part of the soft actuator moving forward. Once the acetone vapor is removed, the deswelling process provides an unbending force (upward bending) that releases the soft actuator back to its original shape. In this situation, the front edge grips the ratcheted substrate and bears a higher frictional force ($F1' > F2'$) accordingly, thus acting as a stationary end that allows forward movement of the back part. Once the recovery motion stops, the actuator is allowed to initiate a new downward and upward bending process after a new acetone absorption/desorption cycle, resulting in a walking soft robot that moves forward a longer distance along the direction of ν, as shown in **Fig. S36**.

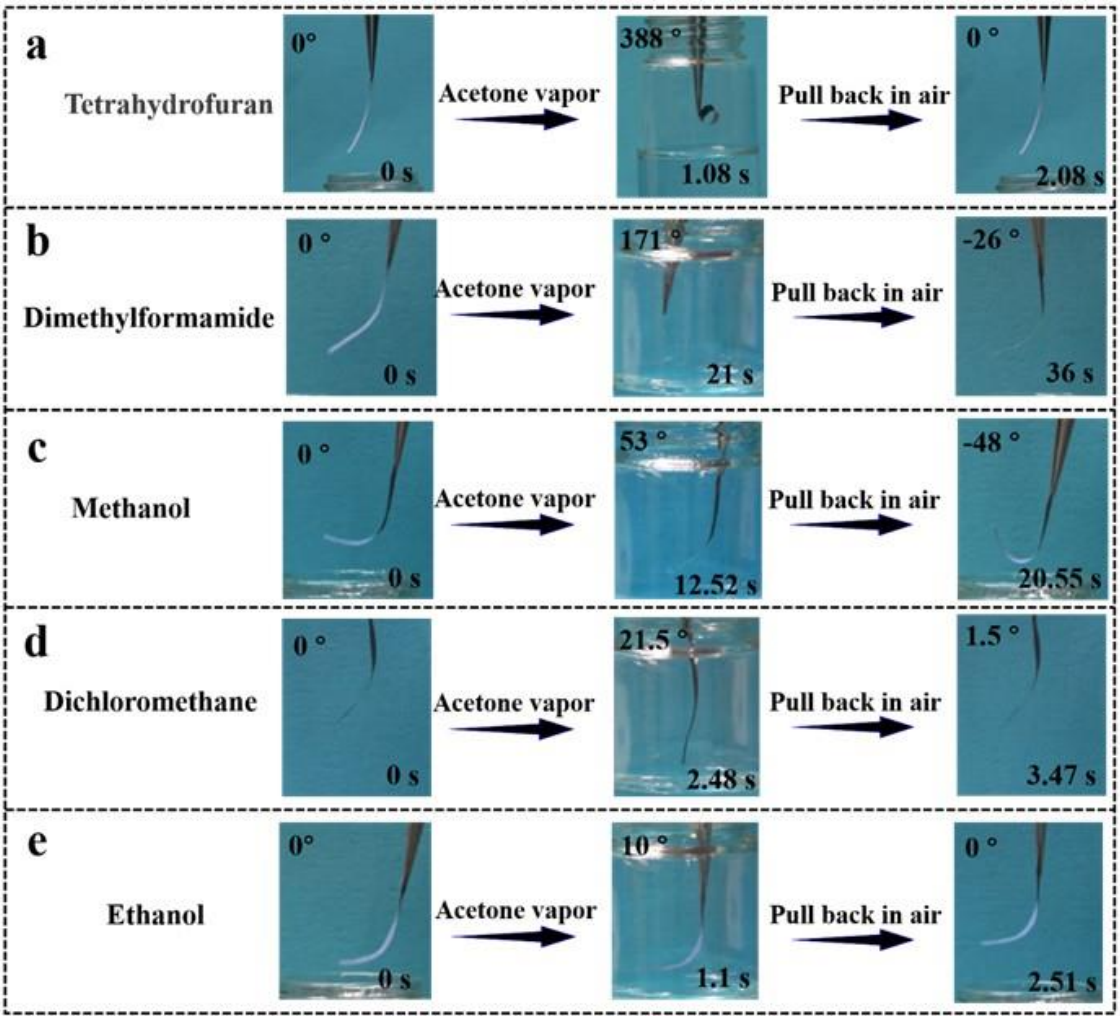

Fig. S33 Photographs of the bending and recovering process of the angle-independent structural color soft actuators in the vapor of different types of solvents

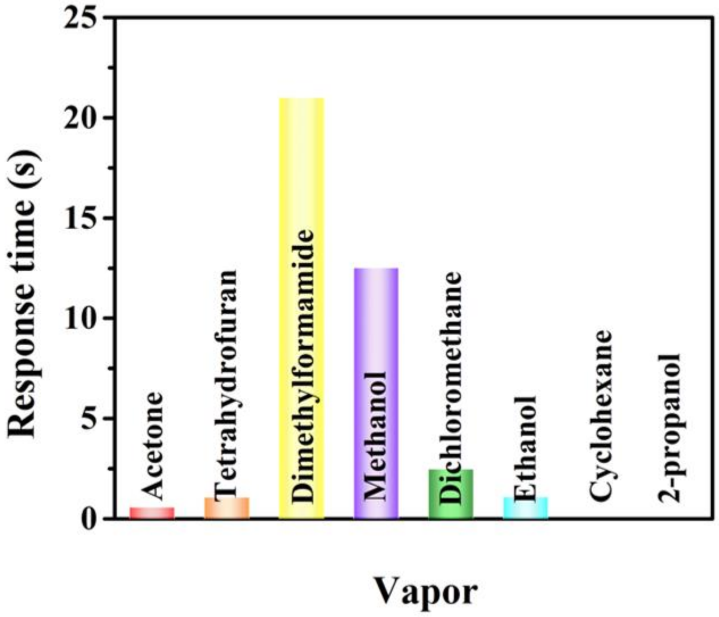

Fig. S34 Dependence of response time of the soft actuator on various organic vapors

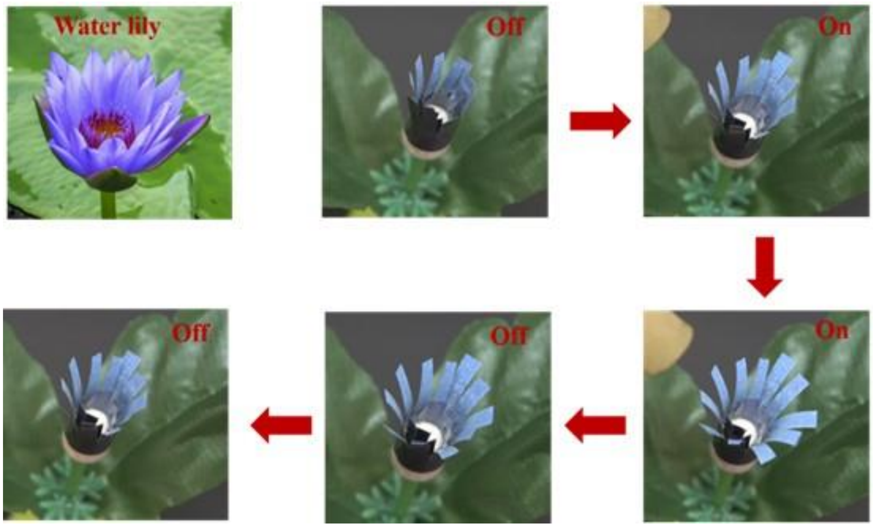

Fig. S35 The process of biomimetic flower in various stages of blooming

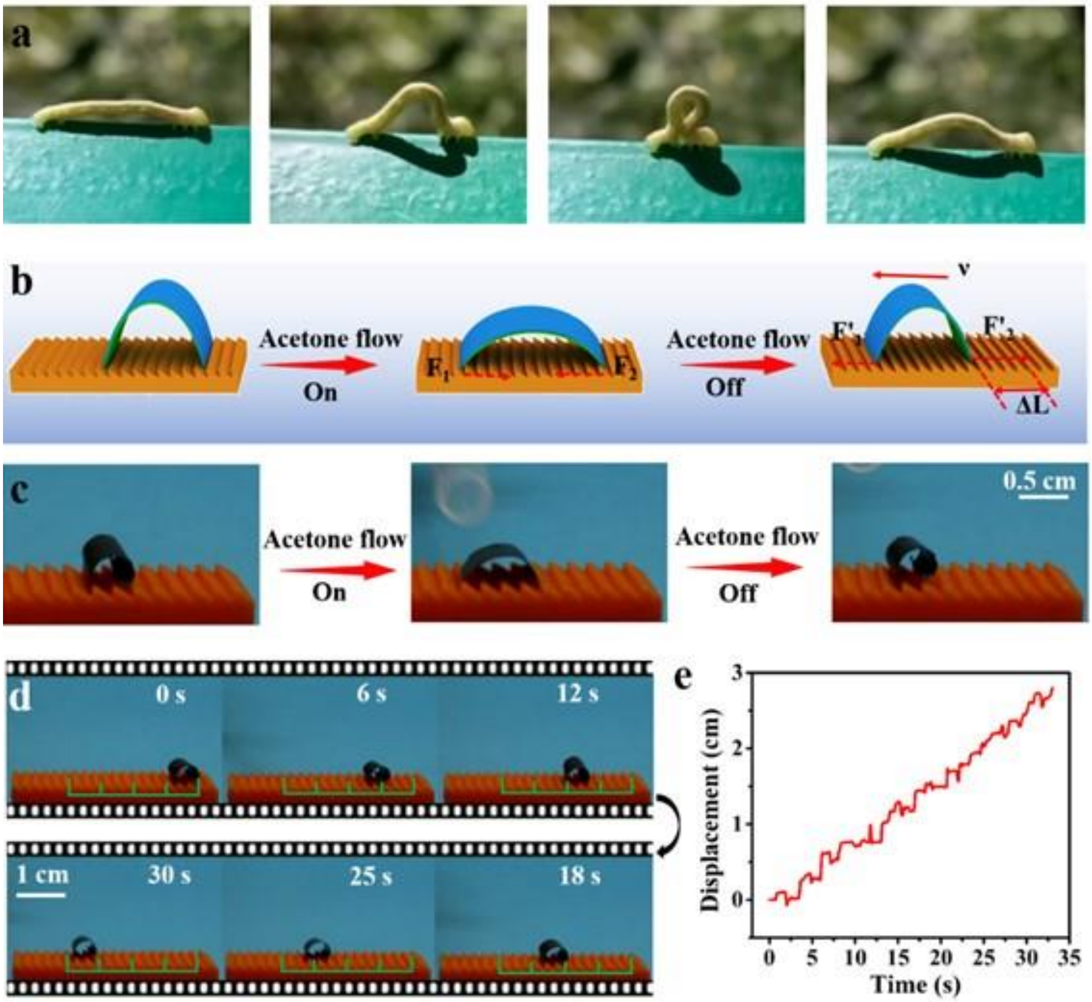

Fig. S36 Biomimetic walking soft robot. (**a**) The movement process of the inchworm in nature. (**b, c**) The schematic diagram of the walking soft robot and its movement. (**d**) The movement process of the walking soft robot under acetone vapor absorption/desorption cycles. (**e**) Horizontal displacement and velocity change of the walking soft robot along the horizontal position over time

Table S1 Summary of different materials used for the design of actuators. Note: In each cited paper, we choose the most rapid speed for comparison with our results

Material	Response type	Response/ Recovery time (s)	Color type	Curvature/ Bending angle (mm^{-1})/(°)	Refs.
MXene/SiO_2@ PVDF	Acetone	1.16/0.24	Non-iridescent structural colors	0.52/360	This work
PNIPAM-K6APA	Temperature	8/211	Fluorescent colors	---/180	Angew. Chem. Int. Ed. **131**, 16243 (2019)
GO-PNIPAM	Temperature	600/---	Fluorescent colors	---/360	Adv. Funct. Mater. **28**, 1704568 (2018)
PNIPAM/ rGO	NIR	120/---	Iridescent structural colors	---/60	Adv. Mater. **29**, 1704569 (2017).
LCN/GO	Visible light	2.94/3.53	Iridescent structural colors	---/97	Adv. Opt. Mater. **6**, 1800131 (2018).
PA/LCN	Humidity	10/---	Fluorescent colors	---/275	Adv. Funct. Mater. **31**, 2010578 (2021).
P(IL-co-MMA)	Humidity	4/---	Iridescent structural colors	2.1/1440	ACS Nano **12**, 12149 (2018).
PTMPTA	Acetone	---	Iridescent structural colors	---	Matter **1**, 626-638 (2019).
PEGDA–PUA	Acetone	15/85	Iridescent structural colors	---/90	J. Mater. Sci. **55**, 817-827 (2019).
PVDF	Acetone	12/---	---	3/---	Adv. Mater. **29**, 1702231 (2017).
PVDF/MS	Acetone	20/---	---	1/---	Mater. Eng. **304**, 1800501 (2019).
GO/CA	Acetone	19/38	---	---/720	Smart Mater. Struct. **28**, 105043 (2019).
cPVA/PVDF/ CNT@PDMS	Acetone	5/25	---	0.65/---	Chem. Eng. J. **394**, 124919 (2020).
PCAD@AG	Humidity	7/33	---	---/260	Nat. Commun. **6**, 7429 (2015).
Ptriaz-TA	Humidity	8/9	---	0.32/---	Nat. Commun. **9**, 1717 (2018).
CNP	Humidity	---	---	---/120	Nat. Mater. **15**, 1084 (2016).

Supplementary References

[S1]J. Li, J. Fan, R. Cao, Z. Zhen, J. Du et al., Encapsulated dye/polymer nanoparticles prepared via miniemulsion polymerization for inkjet printing. ACS Omega **3**(7), 7380-7387 (2018). https://doi.org/10.1021/acsomega.8b01151
